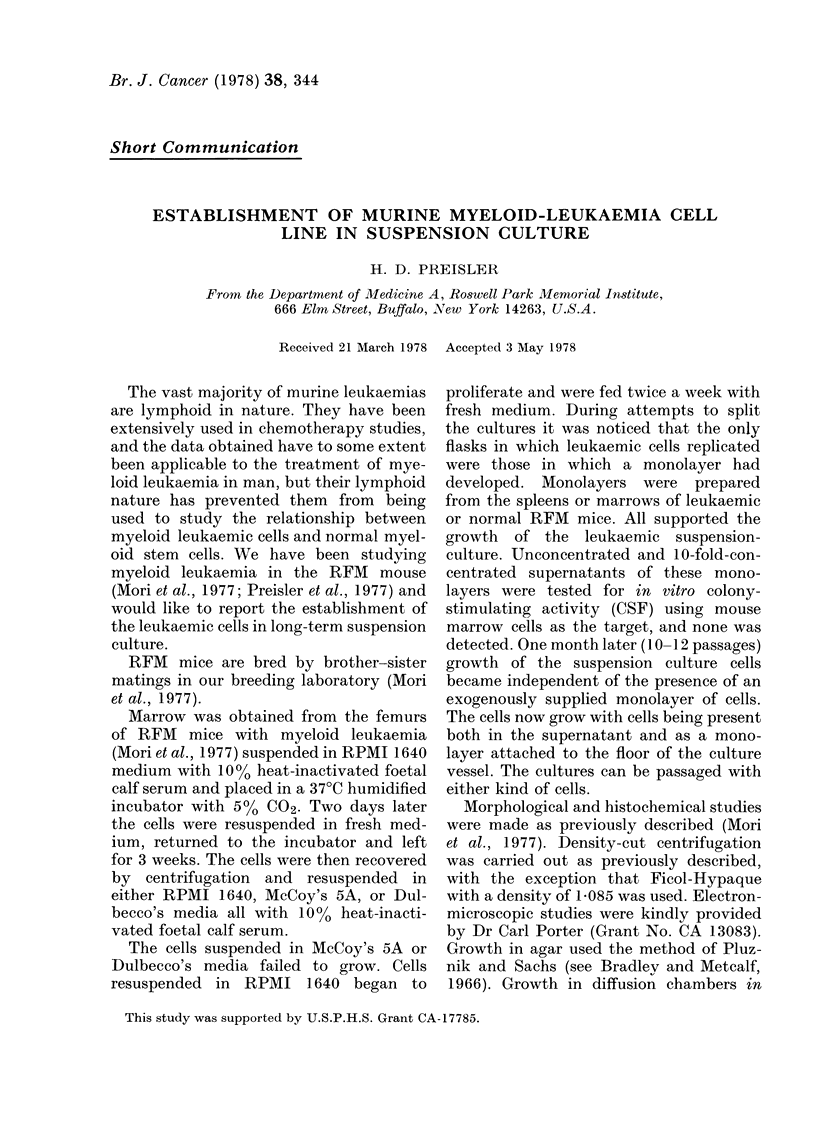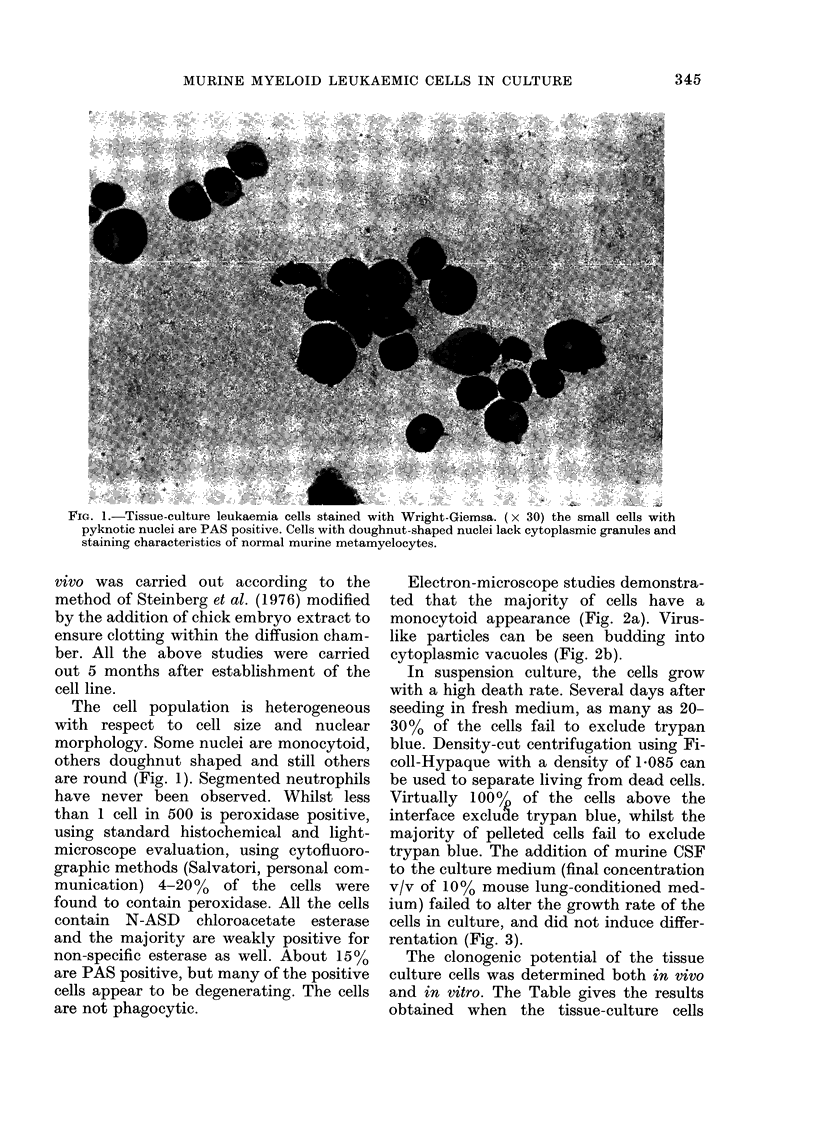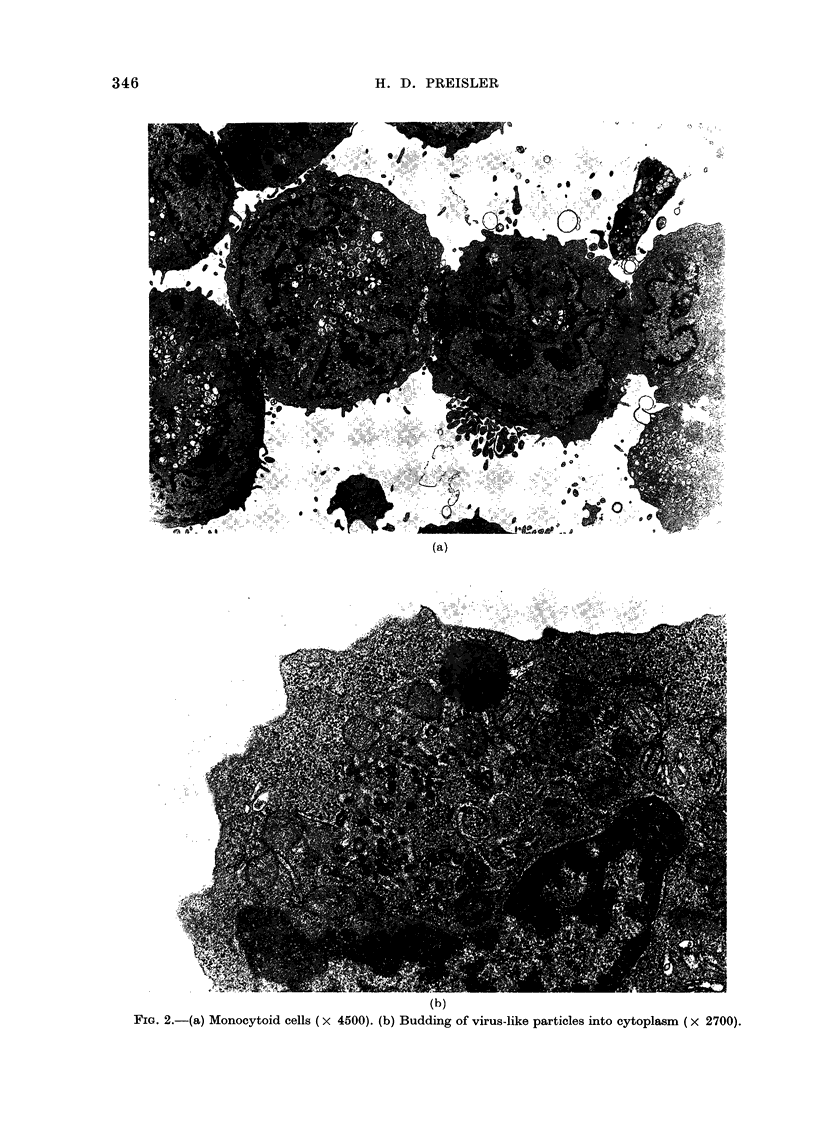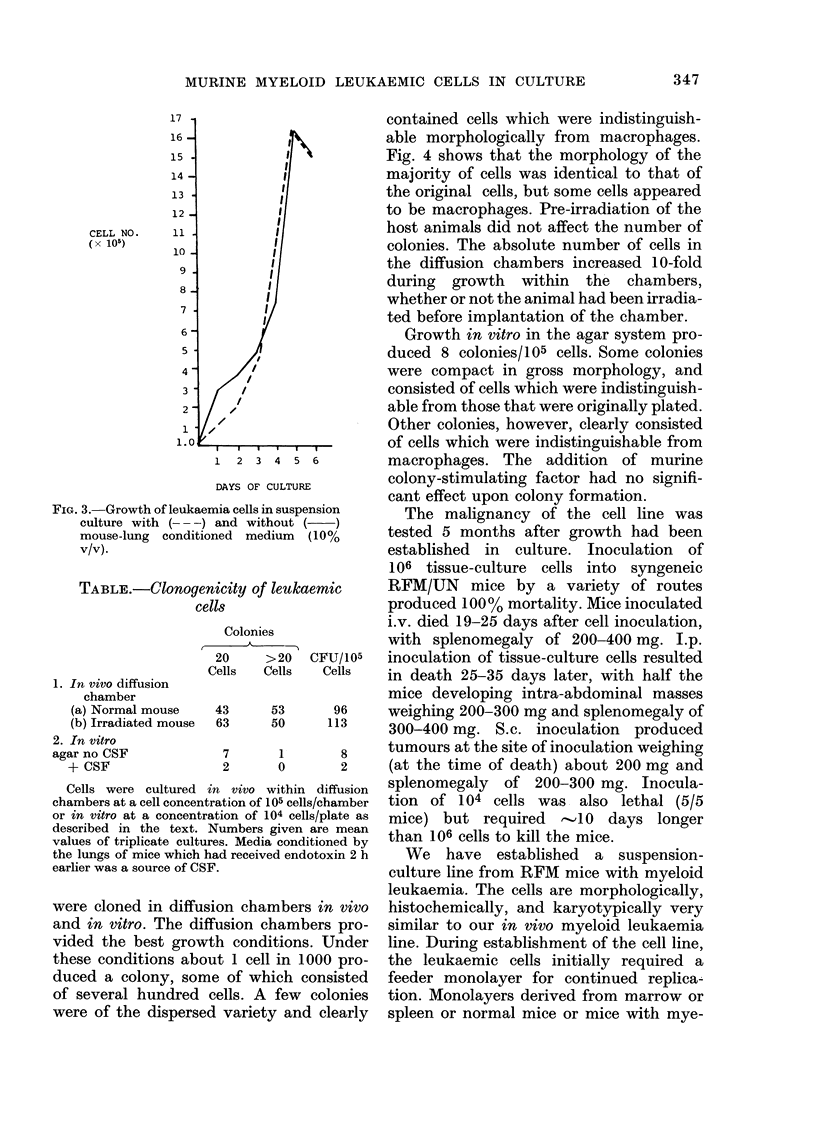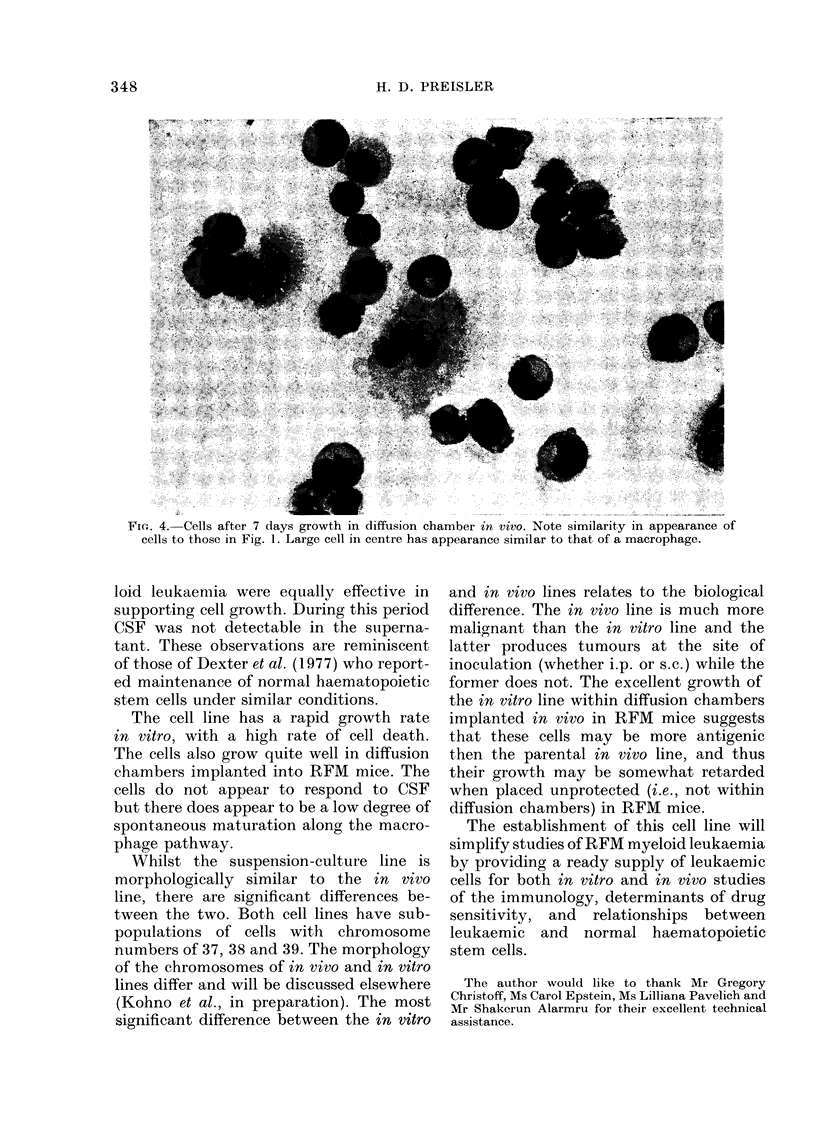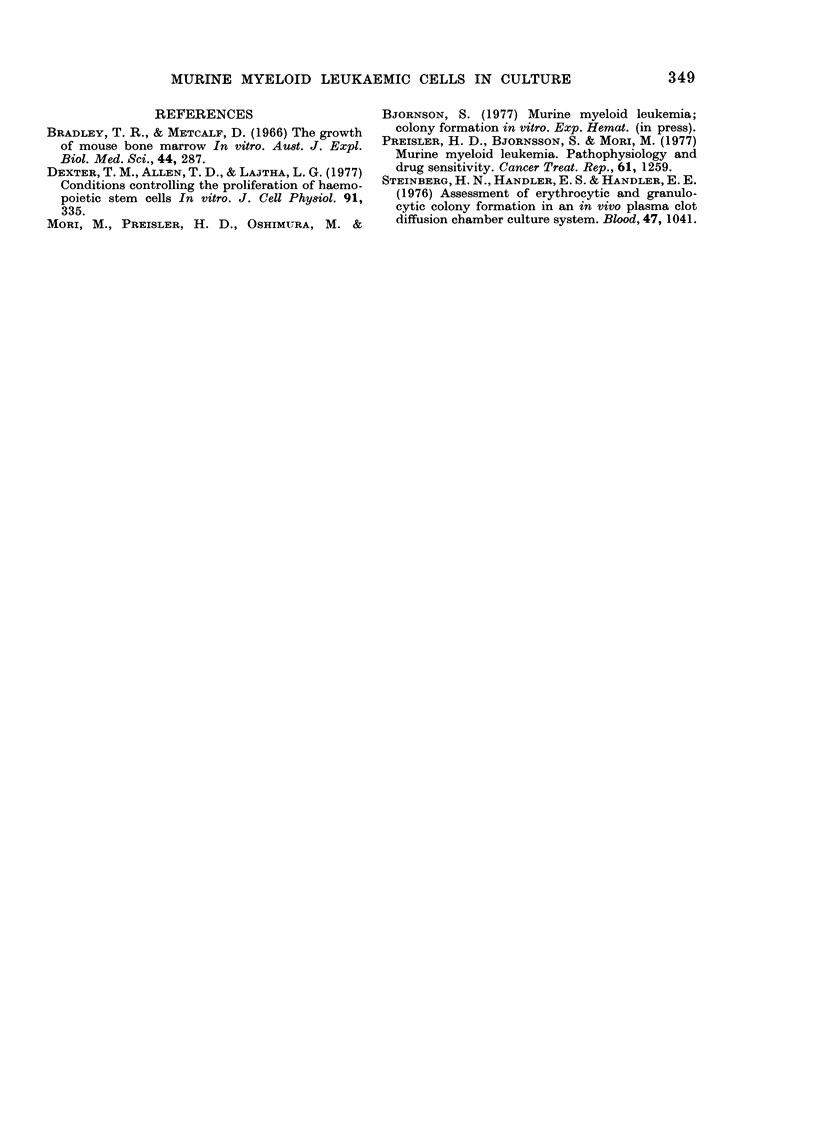# Establishment of murine myeloid-leukaemia cell line in suspension culture.

**DOI:** 10.1038/bjc.1978.209

**Published:** 1978-08

**Authors:** H. D. Preisler

## Abstract

**Images:**


*Br. J. Cancer* (1978) **38**, 344

***Short Communication***

### ESTABLISHMENT OF MURINE MYELOID-LEUKAEMIA CELL LINE IN SUSPENSION CULTURE

H. D. PREISLER

*From the Department of Medicine A, Roswell Park Memorial Institute, 666 Elm Street, Buffalo, New York* 14263, *U.S.A.*

Received 21 March 1978 Accepted 3 May 1978

The vast majority of murine leukaemias are lymphoid in nature. They have been extensively used in chemotherapy studies, and the data obtained have to some extent been applicable to the treatment of myeloid leukaemia in man, but their lymphoid nature has prevented them from being used to study the relationship between myeloid leukaemic cells and normal myeloid stem cells. We have been studying myeloid leukaemia in the RFM mouse (Mori *et al.*, 1977; Preisler *et al.*, 1977) and would like to report the establishment of the leukaemic cells in long-term suspension culture.

RFM mice are bred by brother–sister matings in our breeding laboratory (Mori *et al.*, 1977).

Marrow was obtained from the femurs of RFM mice with myeloid leukaemia (Mori *et al.*, 1977) suspended in RPMI 1640 medium with 10% heat-inactivated foetal calf serum and placed in a 37°C humidified incubator with 5% $CO_2$. Two days later the cells were resuspended in fresh medium, returned to the incubator and left for 3 weeks. The cells were then recovered by centrifugation and resuspended in either RPMI 1640, McCoy's 5A, or Dulbecco's media all with 10% heat-inactivated foetal calf serum.

The cells suspended in McCoy's 5A or Dulbecco's media failed to grow. Cells resuspended in RPMI 1640 began to proliferate and were fed twice a week with fresh medium. During attempts to split the cultures it was noticed that the only flasks in which leukaemic cells replicated were those in which a monolayer had developed. Monolayers were prepared from the spleens or marrows of leukaemic or normal RFM mice. All supported the growth of the leukaemic suspension-culture. Unconcentrated and 10-fold-concentrated supernatants of these monolayers were tested for *in vitro* colony-stimulating activity (CSF) using mouse marrow cells as the target, and none was detected. One month later (10–12 passages) growth of the suspension culture cells became independent of the presence of an exogenously supplied monolayer of cells. The cells now grow with cells being present both in the supernatant and as a monolayer attached to the floor of the culture vessel. The cultures can be passaged with either kind of cells.

Morphological and histochemical studies were made as previously described (Mori *et al.*, 1977). Density-cut centrifugation was carried out as previously described, with the exception that Ficol-Hypaque with a density of 1·085 was used. Electron-microscopic studies were kindly provided by Dr Carl Porter (Grant No. CA 13083). Growth in agar used the method of Pluznik and Sachs (see Bradley and Metcalf, 1966). Growth in diffusion chambers *in*

This study was supported by U.S.P.H.S. Grant CA-17785.

FIG. 1.—Tissue-culture leukaemia cells stained with Wright-Giemsa. (× 30) the small cells with pyknotic nuclei are PAS positive. Cells with doughnut-shaped nuclei lack cytoplasmic granules and staining characteristics of normal murine metamyelocytes.

*vivo* was carried out according to the method of Steinberg *et al.* (1976) modified by the addition of chick embryo extract to ensure clotting within the diffusion chamber. All the above studies were carried out 5 months after establishment of the cell line.

The cell population is heterogeneous with respect to cell size and nuclear morphology. Some nuclei are monocytoid, others doughnut shaped and still others are round (Fig. 1). Segmented neutrophils have never been observed. Whilst less than 1 cell in 500 is peroxidase positive, using standard histochemical and light-microscope evaluation, using cytofluorographic methods (Salvatori, personal communication) 4–20% of the cells were found to contain peroxidase. All the cells contain N-ASD chloroacetate esterase and the majority are weakly positive for non-specific esterase as well. About 15% are PAS positive, but many of the positive cells appear to be degenerating. The cells are not phagocytic.

Electron-microscope studies demonstrated that the majority of cells have a monocytoid appearance (Fig. 2a). Virus-like particles can be seen budding into cytoplasmic vacuoles (Fig. 2b).

In suspension culture, the cells grow with a high death rate. Several days after seeding in fresh medium, as many as 20–30% of the cells fail to exclude trypan blue. Density-cut centrifugation using Ficoll-Hypaque with a density of 1·085 can be used to separate living from dead cells. Virtually 100% of the cells above the interface exclude trypan blue, whilst the majority of pelleted cells fail to exclude trypan blue. The addition of murine CSF to the culture medium (final concentration v/v of 10% mouse lung-conditioned medium) failed to alter the growth rate of the cells in culture, and did not induce differentation (Fig. 3).

The clonogenic potential of the tissue culture cells was determined both *in vivo* and *in vitro*. The Table gives the results obtained when the tissue-culture cells

(a)

(b)

FIG. 2.—(a) Monocytoid cells (× 4500). (b) Budding of virus-like particles into cytoplasm (× 2700).

FIG. 3.—Growth of leukaemia cells in suspension culture with (- - -) and without (——) mouse-lung conditioned medium (10% v/v).

TABLE.—*Clonogenicity of leukaemic cells*

| | Colonies | | |
|---|---|---|---|
| | 20 Cells | >20 Cells | CFU/$10^5$ Cells |
| 1. *In vivo* diffusion chamber | | | |
| (a) Normal mouse | 43 | 53 | 96 |
| (b) Irradiated mouse | 63 | 50 | 113 |
| 2. *In vitro* | | | |
| agar no CSF | 7 | 1 | 8 |
| + CSF | 2 | 0 | 2 |

Cells were cultured *in vivo* within diffusion chambers at a cell concentration of $10^5$ cells/chamber or *in vitro* at a concentration of $10^4$ cells/plate as described in the text. Numbers given are mean values of triplicate cultures. Media conditioned by the lungs of mice which had received endotoxin 2 h earlier was a source of CSF.

were cloned in diffusion chambers *in vivo* and *in vitro*. The diffusion chambers provided the best growth conditions. Under these conditions about 1 cell in 1000 produced a colony, some of which consisted of several hundred cells. A few colonies were of the dispersed variety and clearly contained cells which were indistinguishable morphologically from macrophages. Fig. 4 shows that the morphology of the majority of cells was identical to that of the original cells, but some cells appeared to be macrophages. Pre-irradiation of the host animals did not affect the number of colonies. The absolute number of cells in the diffusion chambers increased 10-fold during growth within the chambers, whether or not the animal had been irradiated before implantation of the chamber.

Growth *in vitro* in the agar system produced 8 colonies/$10^5$ cells. Some colonies were compact in gross morphology, and consisted of cells which were indistinguishable from those that were originally plated. Other colonies, however, clearly consisted of cells which were indistinguishable from macrophages. The addition of murine colony-stimulating factor had no significant effect upon colony formation.

The malignancy of the cell line was tested 5 months after growth had been established in culture. Inoculation of $10^6$ tissue-culture cells into syngeneic RFM/UN mice by a variety of routes produced 100% mortality. Mice inoculated i.v. died 19–25 days after cell inoculation, with splenomegaly of 200–400 mg. I.p. inoculation of tissue-culture cells resulted in death 25–35 days later, with half the mice developing intra-abdominal masses weighing 200–300 mg and splenomegaly of 300–400 mg. S.c. inoculation produced tumours at the site of inoculation weighing (at the time of death) about 200 mg and splenomegaly of 200–300 mg. Inoculation of $10^4$ cells was also lethal (5/5 mice) but required ~10 days longer than $10^6$ cells to kill the mice.

We have established a suspension-culture line from RFM mice with myeloid leukaemia. The cells are morphologically, histochemically, and karyotypically very similar to our *in vivo* myeloid leukaemia line. During establishment of the cell line, the leukaemic cells initially required a feeder monolayer for continued replication. Monolayers derived from marrow or spleen or normal mice or mice with mye-

FIG. 4.—Cells after 7 days growth in diffusion chamber *in vivo*. Note similarity in appearance of cells to those in Fig. 1. Large cell in centre has appearance similar to that of a macrophage.

loid leukaemia were equally effective in supporting cell growth. During this period CSF was not detectable in the supernatant. These observations are reminiscent of those of Dexter *et al.* (1977) who reported maintenance of normal haematopoietic stem cells under similar conditions.

The cell line has a rapid growth rate *in vitro*, with a high rate of cell death. The cells also grow quite well in diffusion chambers implanted into RFM mice. The cells do not appear to respond to CSF but there does appear to be a low degree of spontaneous maturation along the macrophage pathway.

Whilst the suspension-culture line is morphologically similar to the *in vivo* line, there are significant differences between the two. Both cell lines have subpopulations of cells with chromosome numbers of 37, 38 and 39. The morphology of the chromosomes of *in vivo* and *in vitro* lines differ and will be discussed elsewhere (Kohno *et al.*, in preparation). The most significant difference between the *in vitro* and *in vivo* lines relates to the biological difference. The *in vivo* line is much more malignant than the *in vitro* line and the latter produces tumours at the site of inoculation (whether i.p. or s.c.) while the former does not. The excellent growth of the *in vitro* line within diffusion chambers implanted *in vivo* in RFM mice suggests that these cells may be more antigenic then the parental *in vivo* line, and thus their growth may be somewhat retarded when placed unprotected (*i.e.*, not within diffusion chambers) in RFM mice.

The establishment of this cell line will simplify studies of RFM myeloid leukaemia by providing a ready supply of leukaemic cells for both *in vitro* and *in vivo* studies of the immunology, determinants of drug sensitivity, and relationships between leukaemic and normal haematopoietic stem cells.

The author would like to thank Mr Gregory Christoff, Ms Carol Epstein, Ms Lilliana Pavelich and Mr Shakerun Alarmru for their excellent technical assistance.

### REFERENCES

BRADLEY, T. R., & METCALF, D. (1966) The growth of mouse bone marrow *In vitro*. *Aust. J. Expl. Biol. Med. Sci.*, **44**, 287.

DEXTER, T. M., ALLEN, T. D., & LAJTHA, L. G. (1977) Conditions controlling the proliferation of haemopoietic stem cells *In vitro*. *J. Cell Physiol.* **91**, 335.

MORI, M., PREISLER, H. D., OSHIMURA, M. & BJORNSON, S. (1977) Murine myeloid leukemia; colony formation *in vitro*. *Exp. Hemat.* (in press).

PREISLER, H. D., BJORNSSON, S. & MORI, M. (1977) Murine myeloid leukemia. Pathophysiology and drug sensitivity. *Cancer Treat. Rep.*, **61**, 1259.

STEINBERG, H. N., HANDLER, E. S. & HANDLER, E. E. (1976) Assessment of erythrocytic and granulocytic colony formation in an *in vivo* plasma clot diffusion chamber culture system. *Blood*, **47**, 1041.